# *N*-(2-Meth­oxy­phen­yl)benzene­sulfonamide

**DOI:** 10.1107/S1600536810023871

**Published:** 2010-06-26

**Authors:** Aziz-ur-Rehman, Muhammad Arif Sajjad, Mehmet Akkurt, Shahzad Sharif, Muhammad Athar Abbasi, Islam Ullah Khan

**Affiliations:** a Department of Chemistry, Government College University, Lahore 54000, Pakistan; b Department of Physics, Faculty of Arts and Sciences, Erciyes University, 38039 Kayseri, Turkey

## Abstract

The asymmetric unit of the title compound, C_13_H_13_NO_3_S, contains two crystallographically independent mol­ecules in which the dihedral angles between the phenyl and benzene rings are 88.16 (12) and 44.50 (12)°. One of the mol­ecules features an intra­molecular N—H⋯O hydrogen bond. In the crystal, the mol­ecules are linked into dimers by pairs of N—H⋯O hydrogen bonds. The dimers are further connected by C—H⋯O and C—H⋯π inter­actions, forming a three-dimensional network.

## Related literature

For the biological activity of sulfonamides, see: Arshad *et al.* (2008 ▶); Gennarti *et al.* (1994 ▶); Kayser *et al.* (2004 ▶); Rough *et al.* (1998 ▶).
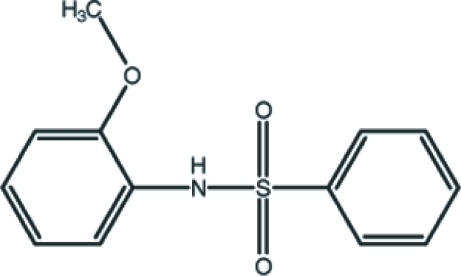


## Experimental

### 

#### Crystal data

C_13_H_13_NO_3_S
*M* *_r_* = 263.31
Monoclinic, 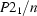

*a* = 8.7705 (2) Å
*b* = 28.1684 (7) Å
*c* = 10.7256 (3) Å
β = 105.968 (1)°
*V* = 2547.53 (11) Å^3^
*Z* = 8
Mo *K*α radiation
μ = 0.25 mm^−1^
*T* = 296 K
0.25 × 0.17 × 0.07 mm

#### Data collection

Bruker APEXII CCD diffractometer
24823 measured reflections
6318 independent reflections
4145 reflections with *I* > 2σ(*I*)
*R*_int_ = 0.043

#### Refinement

*R*[*F* ^2^ > 2σ(*F* ^2^)] = 0.047
*wR*(*F* ^2^) = 0.113
*S* = 1.02
6318 reflections
333 parameters
2 restraints
H atoms treated by a mixture of independent and constrained refinement
Δρ_max_ = 0.29 e Å^−3^
Δρ_min_ = −0.29 e Å^−3^

### 

Data collection: *APEX2* (Bruker, 2007 ▶); cell refinement: *SAINT* (Bruker, 2007 ▶); data reduction: *SAINT*; program(s) used to solve structure: *SHELXS97* (Sheldrick, 2008 ▶); program(s) used to refine structure: *SHELXL97* (Sheldrick, 2008 ▶); molecular graphics: *ORTEP-3* (Farrugia, 1997 ▶); software used to prepare material for publication: *WinGX* (Farrugia, 1999 ▶) and *PLATON* (Spek, 2009 ▶).

## Supplementary Material

Crystal structure: contains datablocks global, I. DOI: 10.1107/S1600536810023871/hb5501sup1.cif

Structure factors: contains datablocks I. DOI: 10.1107/S1600536810023871/hb5501Isup2.hkl

Additional supplementary materials:  crystallographic information; 3D view; checkCIF report

## Figures and Tables

**Table 1:** Hydrogen-bond geometry (Å, °) *Cg*4 is the centroid of the C7′–C12′ phenyl ring.

*D*—H⋯*A*	*D*—H	H⋯*A*	*D*⋯*A*	*D*—H⋯*A*
N1′—H1*N*′⋯O2	0.828 (18)	2.310 (17)	3.074 (2)	153.6 (17)
N1—H1*N*⋯O1′	0.843 (17)	2.129 (17)	2.961 (2)	168.7 (17)
N1—H1*N*⋯O3	0.843 (17)	2.258 (18)	2.592 (2)	103.8 (14)
C4—H4⋯O2′^i^	0.93	2.47	3.377 (3)	167
C4′—H4′⋯*Cg*4^ii^	0.93	2.85	3.601 (2)	138

Symmetry codes: (i) 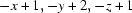; (ii) 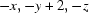.

**Fig. 1.**
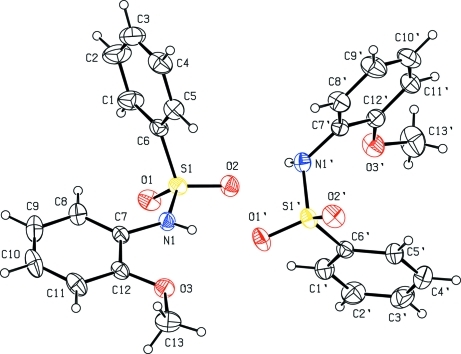
View of the two independent molecules in the asymmetric unit of (I0 with displacement ellipsoids drawn at the 30% probability level.

**Fig. 2.**
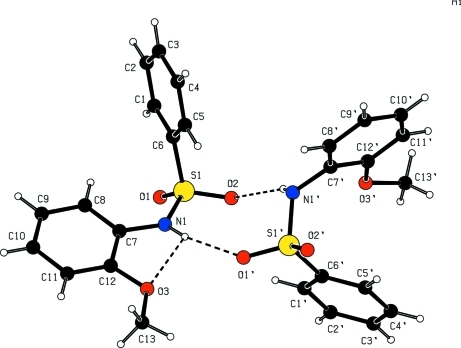
View of N—H···O hydrogen bonds shown as dashed lines between the two independent molecules in the asymmetric unit.

## supplementary crystallographic information

### Comment

Sulfonamides are well known for their enormous potential as biologically active molecules (Rough *et al.*, 1998) in areas such as anti-microbial (Kayser *et al.*, 2004), anti-convulsant (Arshad *et al.*, 2008), anti-cancer agents and for the treatment of inflammatory rheumatic and non-rheumatic processes including onsets and traumatologic lesions (Gennarti *et al.*, 1994). In the present paper, the structure of *N*-(2-methoxyphenyl)benzene sulfonamide has been determined as part of a research program involving the synthesis and biological evaluation of sulfur containing compounds.

In the crystal structure of the title compound (I), (Fig. 1), there exist two independent molecules, A (with S1) and B (with S1'). Both independent molecules are bent at their *S* atoms with the C—S—N(H)—C torsion angles of 67.25 (15)° in molecule A and -81.17 (16)° in molecule B. The dihedral angles between the phenyl and benzene rings is 88.16 (12)° in molecule A and 44.50 (12)° in molecule B.

Molecular packing of (I) is stabilized by N–H···O, C—H···O interactions and C—H···π interactions, forming a three dimensional network (Table 1). Fig. 2 shows N—H···O hydrogen bonds between the molecules A and B in the asymmetric unit.

### Experimental

A mixture benzenesulfonyl chloride (10.0 mmol; 1.45 ml), *ortho*-methoxy aniline (*o-*anisidine) (10.0 mmol; 1.12 ml), aqueous sodium carbonate (10%; 15.0 ml) and water (25 ml) was stirred for one hour at room temperature. The crude mixture was washed with water and dried. The product was dissolved in methanol and recrystallized by slow evaporation of the solvent, to generate colourless blocks of (I) in 74% yield.

### Refinement

The H atoms of the NH groups were located in a difference Fourier map and refined with the N—H distance restrained to 0.86 (2) %A. The other H atoms were positioned geometrically using a riding model with C–H = 0.93 and 0.96 Å. All H atoms were refined with isotropic displacement parameters with *U*_iso_(H) = 1.2*U*_eq_(aromatic, NH) and *U*_iso_(H) = 1.5*U*_eq_(methyl).

### Crystal data

**Table:**

C_13_H_13_NO_3_S	*F*(000) = 1104
*M**_r_* = 263.31	*D*_x_ = 1.373 Mg m^−^^3^
Monoclinic, *P*2_1_/*n*	Mo *K*α radiation, λ = 0.71073 Å
Hall symbol: -P 2yn	Cell parameters from 5117 reflections
*a* = 8.7705 (2) Å	θ = 2.5–23.9°
*b* = 28.1684 (7) Å	µ = 0.25 mm^−^^1^
*c* = 10.7256 (3) Å	*T* = 296 K
β = 105.968 (1)°	Block, colourless
*V* = 2547.53 (11) Å^3^	0.25 × 0.17 × 0.07 mm
*Z* = 8	

### Data collection

**Table:**

Bruker APEXII CCD diffractometer	4145 reflections with *I* > 2σ(*I*)
Radiation source: sealed tube	*R*_int_ = 0.043
graphite	θ_max_ = 28.3°, θ_min_ = 3.3°
φ and ω scans	*h* = −11→11
24823 measured reflections	*k* = −37→37
6318 independent reflections	*l* = −14→14

### Refinement

**Table:**

Refinement on *F*^2^	Primary atom site location: structure-invariant direct methods
Least-squares matrix: full	Secondary atom site location: difference Fourier map
*R*[*F*^2^ > 2σ(*F*^2^)] = 0.047	Hydrogen site location: inferred from neighbouring sites
*wR*(*F*^2^) = 0.113	H atoms treated by a mixture of independent and constrained refinement
*S* = 1.02	*w* = 1/[σ^2^(*F*_o_^2^) + (0.0488*P*)^2^ + 0.3381*P*] where *P* = (*F*_o_^2^ + 2*F*_c_^2^)/3
6318 reflections	(Δ/σ)_max_ = 0.001
333 parameters	Δρ_max_ = 0.29 e Å^−^^3^
2 restraints	Δρ_min_ = −0.29 e Å^−^^3^

### Special details

**Table:**

Geometry. Bond distances, angles *etc*. have been calculated using the rounded fractional coordinates. All su's are estimated from the variances of the (full) variance-covariance matrix. The cell e.s.d.'s are taken into account in the estimation of distances, angles and torsion angles
Refinement. Refinement on *F*^2^ for ALL reflections except those flagged by the user for potential systematic errors. Weighted *R*-factors *wR* and all goodnesses of fit *S* are based on *F*^2^, conventional *R*-factors *R* are based on *F*, with *F* set to zero for negative *F*^2^. The observed criterion of *F*^2^ > σ(*F*^2^) is used only for calculating -*R*-factor-obs *etc*. and is not relevant to the choice of reflections for refinement. *R*-factors based on *F*^2^ are statistically about twice as large as those based on *F*, and *R*-factors based on ALL data will be even larger.

### Fractional atomic coordinates and isotropic or equivalent isotropic displacement parameters (Å^2^)

**Table:**

	*x*	*y*	*z*	*U*_iso_*/*U*_eq_	
S1	0.33115 (5)	0.81887 (2)	0.56961 (5)	0.0412 (2)	
O1	0.29814 (16)	0.77142 (5)	0.59898 (15)	0.0561 (5)	
O2	0.21225 (14)	0.84480 (5)	0.47617 (14)	0.0518 (5)	
O3	0.68975 (15)	0.82034 (5)	0.38159 (14)	0.0507 (5)	
N1	0.48440 (18)	0.81979 (6)	0.51516 (16)	0.0430 (5)	
C1	0.3503 (3)	0.83266 (9)	0.8243 (2)	0.0681 (9)	
C2	0.3878 (4)	0.85873 (11)	0.9379 (3)	0.0865 (11)	
C3	0.4523 (3)	0.90282 (11)	0.9397 (3)	0.0764 (10)	
C4	0.4848 (3)	0.92123 (8)	0.8326 (3)	0.0663 (9)	
C5	0.4496 (2)	0.89536 (7)	0.7189 (2)	0.0511 (7)	
C6	0.3823 (2)	0.85108 (7)	0.71581 (19)	0.0424 (6)	
C7	0.6322 (2)	0.79744 (6)	0.57144 (18)	0.0388 (6)	
C8	0.6717 (3)	0.77602 (7)	0.6914 (2)	0.0536 (7)	
C9	0.8209 (3)	0.75621 (9)	0.7392 (3)	0.0681 (9)	
C10	0.9276 (3)	0.75748 (9)	0.6684 (3)	0.0728 (9)	
C11	0.8897 (2)	0.77854 (8)	0.5478 (3)	0.0596 (8)	
C12	0.7411 (2)	0.79839 (6)	0.4983 (2)	0.0417 (6)	
C13	0.7966 (3)	0.82674 (9)	0.3050 (2)	0.0653 (9)	
S1'	0.31232 (5)	0.92918 (2)	0.25688 (5)	0.0434 (2)	
O1'	0.41671 (16)	0.89021 (5)	0.30204 (16)	0.0599 (5)	
O2'	0.37153 (17)	0.97058 (5)	0.21000 (15)	0.0587 (5)	
O3'	−0.07087 (16)	0.95549 (5)	0.29180 (16)	0.0619 (5)	
N1'	0.24881 (18)	0.94574 (6)	0.37937 (16)	0.0432 (6)	
C1'	0.1023 (3)	0.86096 (7)	0.1393 (2)	0.0540 (7)	
C2'	−0.0250 (3)	0.84437 (9)	0.0434 (3)	0.0675 (9)	
C3'	−0.1041 (3)	0.87391 (10)	−0.0551 (2)	0.0720 (10)	
C4'	−0.0567 (3)	0.92019 (9)	−0.0586 (2)	0.0687 (9)	
C5'	0.0710 (3)	0.93721 (8)	0.0355 (2)	0.0542 (7)	
C6'	0.1504 (2)	0.90740 (7)	0.13445 (18)	0.0412 (6)	
C7'	0.1746 (2)	0.99118 (7)	0.37841 (18)	0.0415 (6)	
C8'	0.2668 (3)	1.02998 (7)	0.4245 (2)	0.0570 (8)	
C9'	0.1992 (3)	1.07399 (8)	0.4279 (3)	0.0711 (10)	
C10'	0.0390 (3)	1.07863 (9)	0.3853 (3)	0.0694 (10)	
C11'	−0.0561 (3)	1.04027 (8)	0.3392 (2)	0.0593 (8)	
C12'	0.0103 (2)	0.99586 (7)	0.33485 (19)	0.0453 (7)	
C13'	−0.2368 (3)	0.95970 (11)	0.2309 (3)	0.0931 (13)	
H1	0.30370	0.80290	0.82150	0.0820*	
H1N	0.476 (2)	0.8381 (6)	0.4514 (17)	0.0520*	
H2	0.36910	0.84620	1.01260	0.1040*	
H3	0.47460	0.92060	1.01560	0.0910*	
H4	0.53060	0.95110	0.83600	0.0800*	
H5	0.47120	0.90770	0.64510	0.0610*	
H8	0.59870	0.77490	0.74000	0.0640*	
H9	0.84830	0.74190	0.82050	0.0820*	
H10	1.02730	0.74400	0.70170	0.0870*	
H11	0.96360	0.77940	0.50010	0.0710*	
H13A	0.88270	0.84670	0.35050	0.0980*	
H13B	0.74210	0.84140	0.22420	0.0980*	
H13C	0.83730	0.79650	0.28840	0.0980*	
H1'	0.15550	0.84110	0.20650	0.0650*	
H1N'	0.210 (2)	0.9225 (6)	0.4068 (19)	0.0520*	
H2'	−0.05770	0.81300	0.04530	0.0810*	
H3'	−0.19020	0.86250	−0.11970	0.0860*	
H4'	−0.11130	0.94010	−0.12520	0.0820*	
H5'	0.10370	0.96850	0.03280	0.0650*	
H8'	0.37640	1.02660	0.45380	0.0680*	
H9'	0.26260	1.10020	0.45910	0.0850*	
H10'	−0.00680	1.10830	0.38740	0.0830*	
H11'	−0.16560	1.04410	0.31080	0.0710*	
H13D	−0.28950	0.97050	0.29310	0.1400*	
H13E	−0.27860	0.92930	0.19770	0.1400*	
H13F	−0.25390	0.98210	0.16090	0.1400*	

### Atomic displacement parameters (Å^2^)

**Table:**

	*U*^11^	*U*^22^	*U*^33^	*U*^12^	*U*^13^	*U*^23^
S1	0.0350 (2)	0.0431 (3)	0.0485 (3)	0.0004 (2)	0.0165 (2)	−0.0006 (2)
O1	0.0577 (9)	0.0437 (8)	0.0721 (11)	−0.0080 (7)	0.0265 (8)	−0.0027 (7)
O2	0.0329 (7)	0.0658 (9)	0.0574 (9)	0.0070 (6)	0.0137 (6)	0.0053 (7)
O3	0.0403 (7)	0.0654 (9)	0.0508 (8)	0.0048 (7)	0.0200 (6)	0.0008 (7)
N1	0.0351 (8)	0.0546 (10)	0.0419 (9)	0.0105 (7)	0.0148 (7)	0.0079 (7)
C1	0.0940 (18)	0.0595 (15)	0.0663 (16)	−0.0063 (13)	0.0479 (14)	−0.0035 (12)
C2	0.114 (2)	0.098 (2)	0.0639 (18)	0.0013 (19)	0.0522 (17)	−0.0102 (16)
C3	0.0722 (17)	0.093 (2)	0.0642 (17)	0.0062 (15)	0.0193 (14)	−0.0275 (15)
C4	0.0516 (13)	0.0576 (15)	0.0858 (19)	−0.0042 (11)	0.0126 (13)	−0.0189 (13)
C5	0.0466 (11)	0.0494 (12)	0.0588 (14)	−0.0019 (10)	0.0169 (10)	0.0001 (10)
C6	0.0410 (10)	0.0436 (11)	0.0469 (11)	0.0050 (8)	0.0195 (9)	0.0004 (9)
C7	0.0333 (9)	0.0354 (10)	0.0463 (11)	0.0035 (8)	0.0084 (8)	−0.0031 (8)
C8	0.0521 (12)	0.0505 (13)	0.0560 (13)	0.0049 (10)	0.0114 (10)	0.0089 (10)
C9	0.0635 (15)	0.0601 (15)	0.0686 (16)	0.0082 (12)	−0.0022 (13)	0.0190 (12)
C10	0.0448 (12)	0.0618 (16)	0.101 (2)	0.0167 (11)	0.0017 (13)	0.0162 (14)
C11	0.0365 (11)	0.0537 (13)	0.0886 (18)	0.0076 (10)	0.0174 (11)	−0.0007 (12)
C12	0.0328 (9)	0.0362 (10)	0.0543 (12)	0.0000 (8)	0.0091 (8)	−0.0062 (9)
C13	0.0596 (13)	0.0753 (16)	0.0730 (16)	−0.0073 (12)	0.0383 (13)	−0.0062 (13)
S1'	0.0351 (2)	0.0419 (3)	0.0543 (3)	0.0050 (2)	0.0143 (2)	0.0066 (2)
O1'	0.0452 (8)	0.0579 (9)	0.0768 (11)	0.0199 (7)	0.0172 (7)	0.0121 (8)
O2'	0.0542 (8)	0.0521 (9)	0.0770 (11)	−0.0079 (7)	0.0304 (8)	0.0062 (8)
O3'	0.0368 (7)	0.0664 (10)	0.0780 (11)	−0.0051 (7)	0.0085 (7)	−0.0066 (8)
N1'	0.0405 (9)	0.0425 (10)	0.0463 (10)	0.0013 (7)	0.0113 (7)	0.0067 (7)
C1'	0.0641 (13)	0.0433 (12)	0.0555 (13)	0.0007 (10)	0.0178 (11)	0.0076 (10)
C2'	0.0769 (16)	0.0555 (15)	0.0686 (16)	−0.0142 (12)	0.0174 (14)	−0.0068 (12)
C3'	0.0709 (16)	0.0831 (19)	0.0549 (15)	−0.0082 (14)	0.0053 (12)	−0.0136 (13)
C4'	0.0729 (16)	0.0741 (17)	0.0503 (14)	0.0100 (13)	0.0023 (12)	0.0064 (12)
C5'	0.0635 (13)	0.0466 (12)	0.0515 (13)	0.0081 (10)	0.0141 (11)	0.0089 (10)
C6'	0.0431 (10)	0.0406 (11)	0.0429 (11)	0.0061 (8)	0.0168 (9)	0.0027 (8)
C7'	0.0403 (10)	0.0456 (11)	0.0391 (10)	0.0035 (8)	0.0120 (8)	0.0001 (8)
C8'	0.0460 (11)	0.0542 (13)	0.0696 (15)	−0.0039 (10)	0.0141 (11)	−0.0114 (11)
C9'	0.0695 (16)	0.0507 (14)	0.094 (2)	−0.0067 (12)	0.0242 (14)	−0.0202 (13)
C10'	0.0766 (17)	0.0563 (15)	0.0807 (18)	0.0155 (13)	0.0306 (14)	−0.0106 (13)
C11'	0.0474 (12)	0.0714 (16)	0.0611 (15)	0.0170 (11)	0.0183 (11)	−0.0006 (12)
C12'	0.0389 (10)	0.0543 (13)	0.0433 (11)	0.0013 (9)	0.0122 (9)	−0.0005 (9)
C13'	0.0414 (13)	0.098 (2)	0.123 (3)	−0.0113 (13)	−0.0060 (14)	−0.0054 (18)

### Geometric parameters (Å, °)

**Table:**

S1—O1	1.4212 (15)	C5—H5	0.9300
S1—O2	1.4319 (15)	C8—H8	0.9300
S1—N1	1.6063 (17)	C9—H9	0.9300
S1—C6	1.760 (2)	C10—H10	0.9300
S1'—C6'	1.7582 (19)	C11—H11	0.9300
S1'—N1'	1.6299 (17)	C13—H13A	0.9600
S1'—O1'	1.4260 (15)	C13—H13C	0.9600
S1'—O2'	1.4235 (15)	C13—H13B	0.9600
O3—C12	1.357 (2)	C1'—C2'	1.375 (4)
O3—C13	1.417 (3)	C1'—C6'	1.380 (3)
O3'—C12'	1.354 (2)	C2'—C3'	1.374 (4)
O3'—C13'	1.427 (3)	C3'—C4'	1.372 (4)
N1—C7	1.418 (2)	C4'—C5'	1.372 (3)
N1—H1N	0.843 (17)	C5'—C6'	1.382 (3)
N1'—C7'	1.435 (3)	C7'—C12'	1.394 (3)
N1'—H1N'	0.828 (18)	C7'—C8'	1.369 (3)
C1—C2	1.383 (4)	C8'—C9'	1.379 (3)
C1—C6	1.372 (3)	C9'—C10'	1.359 (4)
C2—C3	1.363 (4)	C10'—C11'	1.371 (4)
C3—C4	1.360 (4)	C11'—C12'	1.386 (3)
C4—C5	1.381 (4)	C1'—H1'	0.9300
C5—C6	1.376 (3)	C2'—H2'	0.9300
C7—C12	1.393 (3)	C3'—H3'	0.9300
C7—C8	1.376 (3)	C4'—H4'	0.9300
C8—C9	1.385 (4)	C5'—H5'	0.9300
C9—C10	1.358 (4)	C8'—H8'	0.9300
C10—C11	1.378 (4)	C9'—H9'	0.9300
C11—C12	1.383 (3)	C10'—H10'	0.9300
C1—H1	0.9300	C11'—H11'	0.9300
C2—H2	0.9300	C13'—H13D	0.9600
C3—H3	0.9300	C13'—H13E	0.9600
C4—H4	0.9300	C13'—H13F	0.9600
			
O1—S1—O2	118.77 (9)	C9—C10—H10	120.00
O1—S1—N1	109.73 (9)	C11—C10—H10	120.00
O1—S1—C6	107.75 (9)	C10—C11—H11	120.00
O2—S1—N1	104.98 (9)	C12—C11—H11	120.00
O2—S1—C6	108.59 (9)	O3—C13—H13A	109.00
N1—S1—C6	106.38 (9)	H13A—C13—H13C	109.00
O1'—S1'—O2'	119.21 (9)	H13B—C13—H13C	109.00
O1'—S1'—N1'	106.05 (9)	H13A—C13—H13B	109.00
O1'—S1'—C6'	107.24 (9)	O3—C13—H13B	109.00
O2'—S1'—N1'	106.80 (9)	O3—C13—H13C	110.00
O2'—S1'—C6'	108.68 (9)	C2'—C1'—C6'	119.2 (2)
N1'—S1'—C6'	108.48 (9)	C1'—C2'—C3'	120.3 (2)
C12—O3—C13	119.21 (16)	C2'—C3'—C4'	120.3 (2)
C12'—O3'—C13'	117.44 (18)	C3'—C4'—C5'	120.3 (2)
S1—N1—C7	126.62 (14)	C4'—C5'—C6'	119.3 (2)
C7—N1—H1N	118.8 (12)	S1'—C6'—C5'	119.63 (16)
S1—N1—H1N	114.2 (12)	C1'—C6'—C5'	120.68 (19)
S1'—N1'—C7'	120.30 (13)	S1'—C6'—C1'	119.68 (15)
C7'—N1'—H1N'	118.5 (13)	C8'—C7'—C12'	119.96 (19)
S1'—N1'—H1N'	109.0 (13)	N1'—C7'—C8'	119.22 (18)
C2—C1—C6	119.5 (2)	N1'—C7'—C12'	120.80 (17)
C1—C2—C3	119.6 (3)	C7'—C8'—C9'	120.8 (2)
C2—C3—C4	121.2 (3)	C8'—C9'—C10'	119.3 (2)
C3—C4—C5	119.8 (2)	C9'—C10'—C11'	121.0 (2)
C4—C5—C6	119.3 (2)	C10'—C11'—C12'	120.3 (2)
C1—C6—C5	120.58 (19)	C7'—C12'—C11'	118.65 (19)
S1—C6—C1	119.96 (16)	O3'—C12'—C7'	115.67 (17)
S1—C6—C5	119.42 (15)	O3'—C12'—C11'	125.68 (18)
C8—C7—C12	119.97 (19)	C2'—C1'—H1'	120.00
N1—C7—C8	123.93 (19)	C6'—C1'—H1'	120.00
N1—C7—C12	116.10 (16)	C1'—C2'—H2'	120.00
C7—C8—C9	119.5 (2)	C3'—C2'—H2'	120.00
C8—C9—C10	120.6 (3)	C2'—C3'—H3'	120.00
C9—C10—C11	120.7 (3)	C4'—C3'—H3'	120.00
C10—C11—C12	119.5 (2)	C3'—C4'—H4'	120.00
C7—C12—C11	119.7 (2)	C5'—C4'—H4'	120.00
O3—C12—C7	115.04 (16)	C4'—C5'—H5'	120.00
O3—C12—C11	125.23 (19)	C6'—C5'—H5'	120.00
C6—C1—H1	120.00	C7'—C8'—H8'	120.00
C2—C1—H1	120.00	C9'—C8'—H8'	120.00
C3—C2—H2	120.00	C8'—C9'—H9'	120.00
C1—C2—H2	120.00	C10'—C9'—H9'	120.00
C4—C3—H3	119.00	C9'—C10'—H10'	119.00
C2—C3—H3	119.00	C11'—C10'—H10'	120.00
C5—C4—H4	120.00	C10'—C11'—H11'	120.00
C3—C4—H4	120.00	C12'—C11'—H11'	120.00
C4—C5—H5	120.00	O3'—C13'—H13D	109.00
C6—C5—H5	120.00	O3'—C13'—H13E	109.00
C7—C8—H8	120.00	O3'—C13'—H13F	109.00
C9—C8—H8	120.00	H13D—C13'—H13E	109.00
C10—C9—H9	120.00	H13D—C13'—H13F	109.00
C8—C9—H9	120.00	H13E—C13'—H13F	110.00
			
O1—S1—N1—C7	−49.05 (18)	C4—C5—C6—S1	177.86 (17)
O2—S1—N1—C7	−177.75 (15)	C4—C5—C6—C1	0.0 (3)
C6—S1—N1—C7	67.25 (18)	C12—C7—C8—C9	1.0 (3)
O1—S1—C6—C1	−14.8 (2)	N1—C7—C12—O3	−0.7 (2)
O1—S1—C6—C5	167.32 (15)	N1—C7—C8—C9	−178.4 (2)
O2—S1—C6—C1	115.07 (18)	C8—C7—C12—O3	179.93 (17)
O2—S1—C6—C5	−62.83 (17)	C8—C7—C12—C11	−1.2 (3)
N1—S1—C6—C1	−132.41 (18)	N1—C7—C12—C11	178.21 (18)
N1—S1—C6—C5	49.70 (18)	C7—C8—C9—C10	−0.5 (4)
N1'—S1'—C6'—C5'	95.33 (18)	C8—C9—C10—C11	0.1 (4)
O1'—S1'—N1'—C7'	163.92 (14)	C9—C10—C11—C12	−0.3 (4)
O2'—S1'—N1'—C7'	35.80 (17)	C10—C11—C12—C7	0.8 (3)
C6'—S1'—N1'—C7'	−81.17 (16)	C10—C11—C12—O3	179.6 (2)
O1'—S1'—C6'—C1'	30.5 (2)	C6'—C1'—C2'—C3'	−0.7 (4)
O1'—S1'—C6'—C5'	−150.54 (17)	C2'—C1'—C6'—S1'	179.66 (19)
O2'—S1'—C6'—C1'	160.64 (17)	C2'—C1'—C6'—C5'	0.7 (3)
O2'—S1'—C6'—C5'	−20.4 (2)	C1'—C2'—C3'—C4'	0.0 (4)
N1'—S1'—C6'—C1'	−83.61 (19)	C2'—C3'—C4'—C5'	0.7 (4)
C13—O3—C12—C7	174.60 (17)	C3'—C4'—C5'—C6'	−0.6 (4)
C13—O3—C12—C11	−4.2 (3)	C4'—C5'—C6'—S1'	−179.01 (18)
C13'—O3'—C12'—C7'	−172.1 (2)	C4'—C5'—C6'—C1'	−0.1 (3)
C13'—O3'—C12'—C11'	8.2 (3)	N1'—C7'—C8'—C9'	−178.5 (2)
S1—N1—C7—C8	−8.1 (3)	C12'—C7'—C8'—C9'	−0.3 (3)
S1—N1—C7—C12	172.47 (14)	N1'—C7'—C12'—O3'	−1.5 (3)
S1'—N1'—C7'—C8'	−88.1 (2)	N1'—C7'—C12'—C11'	178.22 (18)
S1'—N1'—C7'—C12'	93.8 (2)	C8'—C7'—C12'—O3'	−179.62 (18)
C2—C1—C6—C5	−0.7 (4)	C8'—C7'—C12'—C11'	0.1 (3)
C6—C1—C2—C3	1.6 (4)	C7'—C8'—C9'—C10'	0.3 (4)
C2—C1—C6—S1	−178.6 (2)	C8'—C9'—C10'—C11'	0.1 (4)
C1—C2—C3—C4	−1.9 (5)	C9'—C10'—C11'—C12'	−0.3 (4)
C2—C3—C4—C5	1.2 (4)	C10'—C11'—C12'—O3'	179.9 (2)
C3—C4—C5—C6	−0.2 (4)	C10'—C11'—C12'—C7'	0.2 (3)

### Hydrogen-bond geometry (Å, °)

**Table:**

Cg4 is the centroid of the C7'–C12' phenyl ring.

**Table:**

*D*—H···*A*	*D*—H	H···*A*	*D*···*A*	*D*—H···*A*
N1'—H1N'···O2	0.828 (18)	2.310 (17)	3.074 (2)	153.6 (17)
N1—H1N···O1'	0.843 (17)	2.129 (17)	2.961 (2)	168.7 (17)
N1—H1N···O3	0.843 (17)	2.258 (18)	2.592 (2)	103.8 (14)
C4—H4···O2'^i^	0.93	2.47	3.377 (3)	167
C4'—H4'···Cg4^ii^	0.93	2.85	3.601 (2)	138

Symmetry codes: (i) −*x*+1, −*y*+2, −*z*+1; (ii) −*x*, −*y*+2, −*z*.

## References

[bb1] Arshad, M. N., Khan, I. U. & Zia-ur-Rehman, M. (2008). *Acta Cryst.* E**64**, o2283–o2284. 10.1107/S1600536808035721 PMC2959830 21581263

[bb2] Bruker (2007). *APEX2* and *SAINT* Bruker AXS Inc., Madison, Wisconsin, USA.

[bb3] Farrugia, L. J. (1997). *J. Appl. Cryst.* **30**, 565.

[bb4] Farrugia, L. J. (1999). *J. Appl. Cryst.* **32**, 837–838.

[bb5] Gennarti, C., Salom, B., Potenza, D. & Williams, A. (1994). Angew. *Chem. Int. Ed. Engl.* **33**, 2067–2069.

[bb6] Kayser, F. H., Bienz, K. A., Eckert, J. & Zinkernagel, R. M. (2004). *Medical Microbiology*, pp. 1–20. Berlin: Thieme Medical.

[bb7] Rough, W. R., Gwaltney, S. L., Cheng, J., Scheidt, K. A., Mc Kerrow, J. H. & Hansell, E. (1998). *J. Am. Chem. Soc.* **120**, 10994–10995.

[bb8] Sheldrick, G. M. (2008). *Acta Cryst.* A**64**, 112–122. 10.1107/S0108767307043930 18156677

[bb9] Spek, A. L. (2009). *Acta Cryst.* D**65**, 148–155. 10.1107/S090744490804362X PMC2631630 19171970

